# High-throughput RNAi screening for novel modulators of vimentin expression identifies MTHFD2 as a regulator of breast cancer cell migration and invasion

**DOI:** 10.18632/oncotarget.756

**Published:** 2012-12-17

**Authors:** Laura Lehtinen, Kirsi Ketola, Rami Mäkelä, John-Patrick Mpindi, Miro Viitala, Olli Kallioniemi, Kristiina Iljin

**Affiliations:** ^1^ Medical Biotechnology, VTT Technical Research Centre of Finland and Turku Centre for Biotechnology, University of Turku, Turku, Finland; ^2^ Institute for Molecular Medicine Finland (FIMM), University of Helsinki, Finland

**Keywords:** Breast cancer, vimentin, MTHFD2

## Abstract

Vimentin is an intermediate filament protein, with a key role in the epithelial to mesenchymal transition as well as cell invasion, and it is often upregulated during cancer progression. However, relatively little is known about its regulation in cancer cells. Here, we performed an RNA interference screen followed by protein lysate microarray analysis in bone metastatic MDA-MB-231(SA) breast cancer cells to identify novel regulators of vimentin expression. Out of the 596 genes investigated, three novel vimentin regulators EPHB4, WIPF2 and MTHFD2 were identified. The reduced vimentin expression in response to EPHB4, WIPF2 and MTHFD2 silencing was observed at mRNA and protein levels. Bioinformatic analysis of gene expression data across cancers indicated overexpression of EPHB4 and MTHFD2 in breast cancer and high expression associated with poor clinical characteristics. Analysis of 96 cDNA samples derived from both normal and malignant human tissues suggested putative association with metastatic disease. MTHFD2 knockdown resulted in impaired cell migration and invasion into extracellular matrix as well as decreased the fraction of cells with a high CD44 expression, a marker of cancer stem cells. Furthermore, MTHFD2 expression was induced in response to TGF-&beta; stimulation in breast cancer cells. Our results show that MTHFD2 is overexpressed in breast cancer, associates with poor clinical characteristics and promotes cellular features connected with metastatic disease, thus implicating MTHFD2 as a potential drug target to block breast cancer cell migration and invasion.

## INTRODUCTION

Vimentin is a ubiquitous intermediate filament (IF) protein normally expressed in cells of mesenchymal origin [1]. Intermediate filaments along with microtubules and actin microfilaments are important structural components of the cell cytoskeleton. Vimentin has been suggested to be the most widely expressed IF protein in mammals [2]. In addition to mesenchymal cells, vimentin is also expressed by migratory epithelial cells involved in embryonic processes, organogenesis, wound healing and tumor progression [3, 4]. Upregulation of vimentin has been reported in various cancers and aggressive breast cancer cell lines [5-8]. Furthermore, vimentin expression has been associated with poor outcome in breast cancer [9]. Vimentin is a well-known marker of epithelial-mesenchymal transition (EMT), a process which plays a crucial role in carcinogenesis by enabling cancer cell dissemination from metastatic tumors, promoting cell invasion as well as acquisition of therapeutic resistance [10, 11]. During EMT, cells lose epithelial characteristics, such as expression of keratins and E-cadherin and gain mesenchymal properties, such as expression of vimentin and N-cadherin.

In addition to providing a structural scaffold to the cell, intermediate filaments control cell motility. Various reports show that vimentin plays an important role in cell migration [12-14]. Vimentin has also been indicated in regulation of cell survival, cell adhesion and lipid transport [15-17]. Inhibition of vimentin filament integrity causes mesenchymal cells to adopt an epithelial shape [17] and downregulation of vimentin expression has been shown to impair carcinoma cell migration and adhesion [16]. Furthermore, transfection of vimentin antisense oligonucleotide into invasive and vimentin-positive MDA-MB-231 breast cancer cells led to significant decrease of in vitro invasive properties, indicating a functional role for vimentin in regulating cancer cell invasion [18]. Since vimentin has such an important role in regulating cancer cell migration and invasion, it has been suggested as a potential target for cancer therapy [19].

Despite the important role of vimentin in cancer cell behavior, relatively little is known about its regulation. Vimentin has been suggested to be regulated by several factors, including nuclear factor-KappaB (NF-κB), β-catenin/TCF, and transforming growth factor-β (TGF-β), which all have been associated with the EMT-process [6, 20-23]. TGF-β is known to enhance cell migration in mammary epithelial cells [24]. Wu et al. located a TGF-β1 responsive element in the vimentin promoter region and showed that TGF-β regulates vimentin expression via Smads, AP-1 and Sp1 in myoblasts and myotubes during differentiation [21]. Furthermore, Axl receptor tyrosine kinase has been shown to mediate the vimentin-induced effects on cell motility and invasion [25].

Here, we identified three novel vimentin regulating genes in high-throughput RNAi lysate microarray screens in metastatic basal like breast cancer cells. Further *in silico* and *in vitro* validation in cultured breast cancer cells as well as in clinical breast cancer samples, indicated one of these genes, MTHFD2 (methylenetetrahydrofolate dehydrogenase 2), as a potential drug target to block breast cancer cell migration and invasion.

## RESULTS

### RNAi screen identified putative vimentin expression decreasing siRNAs

**Figure 1:**
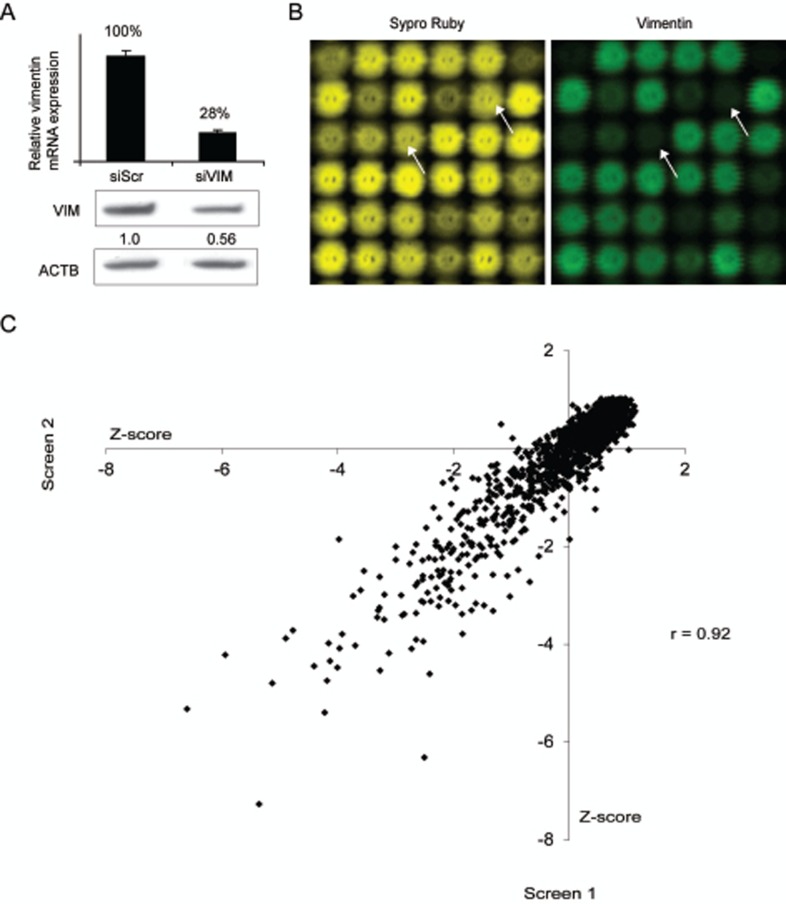
High-throughput RNAi screen to identify vimentin expression regulating siRNAs A: The silencing efficacy of the vimentin targeting siRNA used as a positive control as well as the specificity of the vimentin antibody used in the LMA analysis, was confirmed in MDA-MB-231(SA) cells with qRT-PCR and Western blotting methods. B: LMA slides were stained for total protein (Sypro Ruby, yellow) and for vimentin expression (vimentin, green). Lysate spots derived from vimentin targeting siRNA (indicated with arrows) show decrease in vimentin but not total protein expression. C: A correlation plot (vimentin signal Z-scores) of the two replicate RNAi screens in MDA-MB-231(SA) cells.

Despite the strong evidence of the importance of vimentin in carcinogenesis, its regulation has not been thoroughly investigated. To reveal novel modulators for vimentin expression in breast cancer cells, we conducted two replicate RNAi screens in basal highly metastatic MDA-MB-231(SA) cells. The siRNA library consisted of 2024 siRNAs targeting 596 genes either highly expressed in breast or prostate cancer samples, previously associated with metastasis or over-expressed in bone metastatic vs. parental MDA-MB-231 breast cancer cells [26]. The effect of target gene silencing on vimentin expression was assessed using lysate microarray technology [27]. The integrity of the control siRNAs was validated both at mRNA and protein level (Fig 1A). To exclude siRNAs reducing vimentin expression due to decreased cell proliferation, total protein amount of each spot was measured (Fig 1B). The correlation between two replicate screens was r=0.92, confirming the functionality of the assay (Fig 1C). Hit selection for vimentin expression decreasing siRNAs was done based on the Z-score values (≤ -2, measuring standard deviations from the mean) and amount of total protein in the spot (≥ 0.75). The siRNAs qualified for the hit selection criteria in both or either of the screens are presented in Table 1. All positive control siRNAs targeting *VIM* (n=5) were among these siRNAs.

**Table 1:** Screen results in MDA-MB-231(SA) cells

Screen 1			
Name	Sypro net	VIM net	VIM ratio Z-score
WIPF2	0.895	0.262	−2.617
EPHB4	0.843	0.254	−2.549
VIM siRNA	0.864	0.274	−2.424
VIM siRNA	0.940	0.314	−2.306
VIM siRNA	1.015	0.354	−2.201
VIM siRNA	0.928	0.325	−2.192
VIM siRNA	0.757	0.267	−2.174
MTHFD2	0.769	0.280	−2.093

Screen 2			
Name	Sypro net	VIM net	VIM ratio Z-score
MTHFD2	0.773	0.209	−2.807
VIM siRNA	0.769	0.217	−2.708
VIM siRNA	0.848	0.253	−2.572
VIM siRNA	0.853	0.259	−2.531
VIM siRNA	0.803	0.276	−2.235
VIM siRNA	0.952	0.327	−2.235

### Hit validation

Three siRNA target genes *WIPF2, MTHFD2* and *EPHB4* were selected for further analyzes. None of these genes have been previously described as modulators of vimentin expression in breast cancer.

WIPF2 (WAS/WASL interacting protein family, member 2) belongs to the family of Wiskott-Aldrich syndrome proteins (WASP), shown to participate in WASP-mediated organization of the actin cytoskeleton [28]. MTHFD2 (methylenetetrahydrofolate dehydrogenase 2) is a mitochondrial enzyme with methylenetetrahydrofolate dehydrogenase and methenyltetrahydrofolate cyclohydrolase activities. In normal tissues, MTHFD2 is expressed only during development and it participates in formylmethionyl transfer RNA required for the initiation of protein synthesis in mitochondria [29]. Elevated expression of MTHFD2 has been previously associated with increased risk of bladder cancer [30]. EPHB4 (ephrin type-B receptor 4) belongs to a family of ephrin binding receptors. Overexpression of EPHB4 in breast cancer has been previously described and implicated as promoter of cell migration and invasion as well as a survival factor in breast cancer [21, 31, 32].

**Figure 2:**
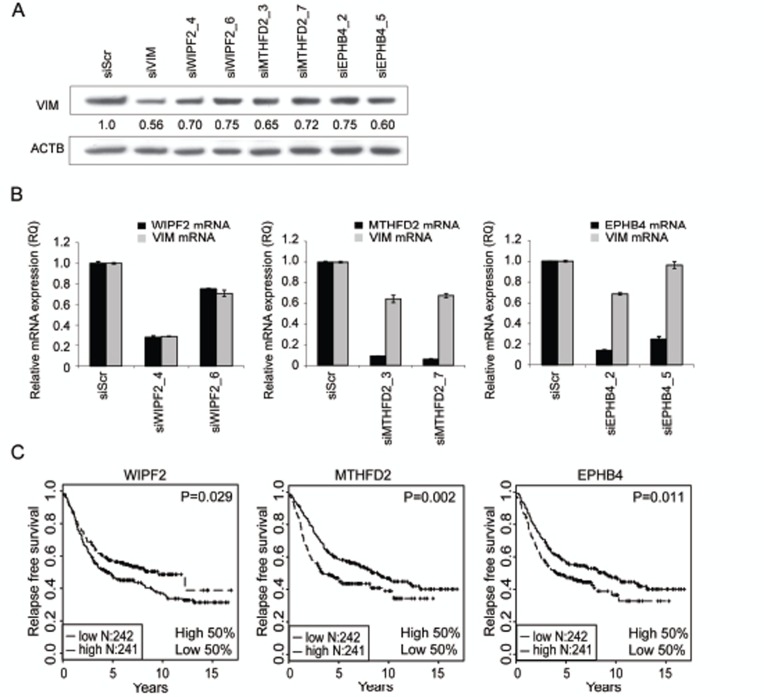
Hit validation A-B: Validation of vimentin protein expression (A) and mRNA expression as well as target gene mRNA expression (B) in MDA-MB-231(SA) cells in response to treatment with WIPF2, EPHB4 or MTHFD2 siRNAs. Two most efficient siRNAs per gene were selected for further analysis. C: Kaplan-Maier plots of WIPF2, EPHB4 and MTHFD2 mRNA expression in breast cancer.

Four siRNAs per gene targeting *WIPF2, MTHFD2* or *EPHB4* were introduced to MDA-MB-231(SA) cells and vimentin protein expression was analyzed 120 hours after transfection (Fig 2A). For each target, two siRNAs reducing vimentin protein expression by at least 25% were selected for further validation. Target gene and vimentin silencing was validated with qRT-PCR (Fig 2B). All siRNAs except WIPF2 targeting siRNA_6 reduced the target gene mRNA expression by at least 70% compared to scrambled control. In addition, all studied siRNAs except siEPHB4_5 reduced the expression of vimentin mRNA by at least 29%. EPHB4 and MTHFD2 target gene silencing was also validated on protein level (Supplementary Figure S1). These results suggest that WIPF2, MTHFD2, and EPHB4 regulate vimentin expression on both protein and mRNA levels.

### Evaluation of prognostic significance of VIM regulators in silico

To analyze the *in vivo* relevance of the identified vimentin regulators, published gene expression data from normal and malignant human tissue samples was utilized. Results from a Kaplan-Meier survival analysis indicated that high expression of MTHFD2 or EPHB4 associate with poor relapse free survival (p=0.002 and p=0.011 respectively) (Fig 2C). On the contrary, high expression of WIPF2 was associated with better relapse free survival (p=0.029). A multivariate analysis of MTHFD2 expression in the same dataset showed that tumor grade was an independent predictor of survival (data not shown), whereas MTHFD2 expression alone was not sufficient to predict prognosis. Based on these results and the fact that EPHB4 had already been described as a target for breast cancer treatment [33], MTHFD2 was chosen for more detailed functional studies.

### MTHFD2 mRNA expression in clinical breast cancer samples

**Figure 3:**
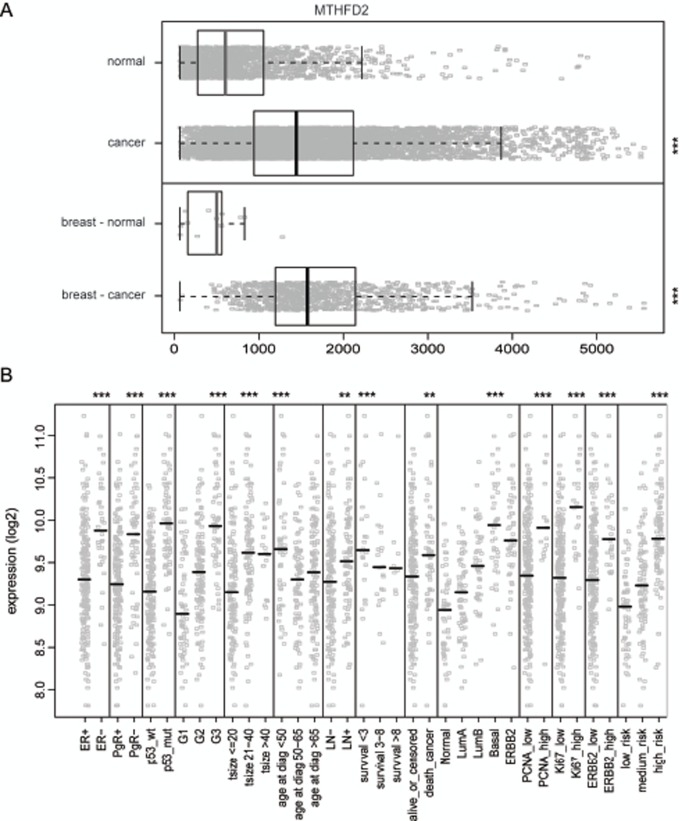
*In silico* gene-expression analysis of MTHFD2 in clinical breast cancer samples A: Box plot analysis of normalized expression values for MTHFD2 mRNA expression in all healthy (n=1659) and malignant tissues (n=5644, ***p < 0.005), as well as in healthy (n=13) and malignant breast tissues (n=957), ***p < 0.005). The box refers to quartile distribution (25-75%) range, with the median shown as a vertical line (grey for normal samples, black for malignant). Data observations which lie more than 1.5*inter-quartile range higher than third quartile, are considered as outliers and are indicated separately. B: Analysis of the association of MTHFD2 mRNA expression with several clinico-phenotypical features in malignant breast tissues. Median value is indicated with a vertical line. The statistically significant p-values are indicated with asterisks (* P<0.05; ** P<0.01; *** P<0.005).

To get an overview of MTHFD2 expression in normal and malignant human breast tissues, a detailed *in silico* analysis of MTHFD2 mRNA expression was performed. The results indicated that in normal tissues MTHFD2 is expressed especially in blood myeloid cells as well as in hematopoietic and mesenchymal stem cells whereas in cancer samples, the highest expression was seen in lymphoma and neuroblastoma (Supplementary Figure S2). Importantly, MTHFD2 mRNA was overexpressed (p<0.005) in clinical breast cancer samples compared to normal breast tissues (Fig. 3A). The association of MTHFD2 mRNA expression to clinical and phenotypical parameters was also analyzed. High MTHFD2 was associated with hormone receptor (ER, PgR) negative, HER2 positive breast cancer as well as with several other characteristics of poor clinical outcome such as p53 mutation, high grade tumors, lymph node (LN) positivity, increased risk of cancer death, basal subtype, increased proliferation measured by Ki67 and PCNA positivity (Fig. 3B). Results of all comparisons are described in Supplementary Table S1.

### MTHFD2 is overexpressed in breast cancer and associates with cancer metastasis

**Figure 4:**
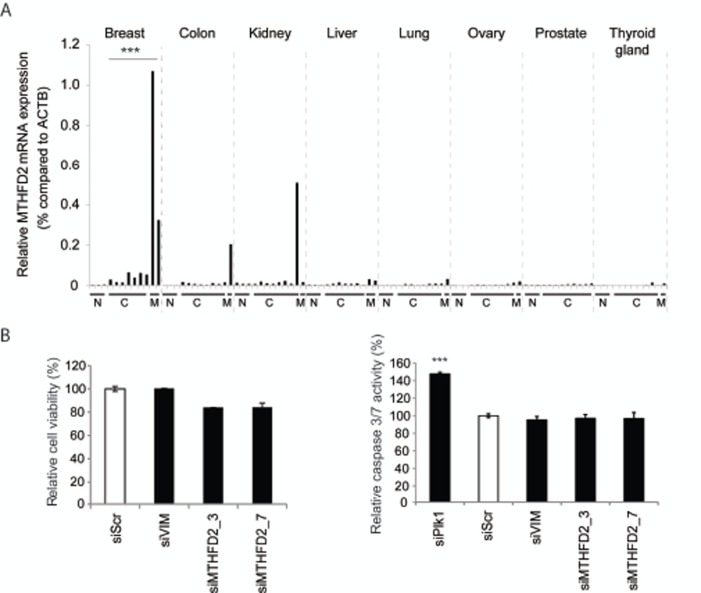
Validation of MTHFD2 mRNA expression in clinical samples and analysis of cell viability and induction of apoptosis in response to MTHFD2 depletion A: A qRT-PCR analysis of MTHFD2 mRNA expression in a human cancer cDNA panel containing samples from normal (N), malignant (C) and metastases (M) from several human cancers. The MTHFD2 expression values were normalized to corresponding β-actin mRNA expression (ACTB) in each sample. B: Analysis of the effect of MTHFD2 depletion on cell viability and induction of apoptosis (caspase 3/7 activity) in MDA-MB-231(SA) cells. The statistically significant p-values are indicated with asterisks (* P<0.05; ** P<0.01; *** P<0.005).

To further investigate MTHFD2 mRNA expression in breast cancer and to study tissue type selectivity, a Tissue Scan cDNA panel was used. The results suggested that MTHFD2 mRNA is overexpressed in breast cancer samples compared to normal breast tissues [Fig. 4A]. In addition to breast cancer, elevated MTHFD2 levels were detected also in colon cancer, kidney cancer and liver cancer samples. Interestingly, MTHFD2 mRNA was expressed at high levels especially in samples derived from metastases.

### *In silico* co-expression analysis of MTHFD2 in clinical breast cancer samples

To investigate the function of MTHFD2 in breast cancer, *in silico* co-expression signatures in clinical breast cancer samples were analyzed [Supplementary Tables S2 and S3]. The results showed that MTHFD2 is expressed in the same samples as genes involved in regulation of cell cycle, cellular movement as well as cellular assembly and organization processes. Genetic disorder, neurological disease and cancer were among the top diseases associated with these genes.

### MTHFD2 knockdown does not significantly alter cell proliferation

The co-expression analysis indicated that MTHFD2 associates with processes controlling cell cycle. To validate whether MTHFD2 knockdown directly affects cell proliferation or induction of apoptosis, MDA-MB-231(SA) cells were transfected as described and assayed for cell viability and caspase 3/7 activity. The results indicated that MTHFD2 depletion did not have a significant effect on cell proliferation or induction of apoptosis in these breast cancer cells (Fig 4B). A Plk1 targeting siRNA was used as a positive control in the apoptosis assay.

### MTHFD2 knockdown inhibits breast cancer cell migration and invasion

**Figure 5:**
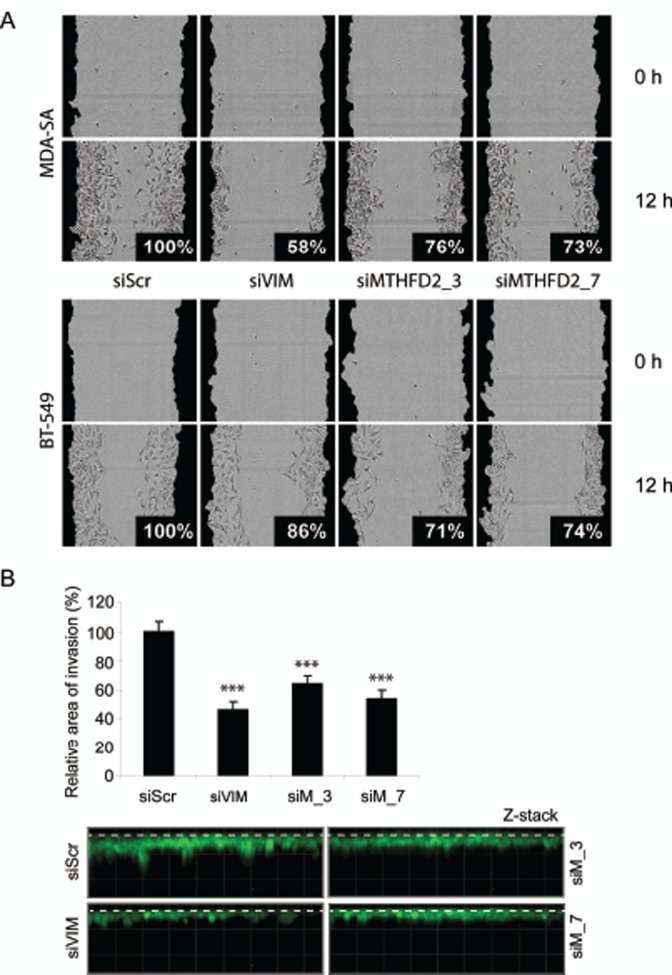
MTHFD2 knockdown reduces breast cancer cell migration and invasion to extracellular matrix A: The effect of MTHFD2 siRNAs on MDA-MB-231(SA) and BT-549 cell motility in wound healing experiment. Cells were automatically imaged once every hour after wound scratching. Wound closure effect was calculated as wound confluence which the cells gained in twelve hours. B: MDA-MB-231(SA) cells transfected with either vimentin targeting siRNA (siVIM), MTHFD2 targeting siRNA (siMTHFD2) or negative control siRNA (siScr), allowed to invade into Matrigel matrix for 4 days and imaged. Shown are the quantifications of two replicate experiments (imaged at 5-10 different positions; ***p < 0.005) and representative images. The dashed lines indicate the top of the Matrigel matrix.

Vimentin is a regulator of cell migration and invasion in breast cancer cells [25]. To validate the defect in cell migration in response to MTHFD2 knockdown, a wound healing assay was done in MDA-MB-231(SA), BT-549 and parental MDA-MB-231 cells. These cell lines were chosen based on mutual expression of MTHFD2 and vimentin mRNAs (Supplementary Figure S3A). In addition to MDA-MB-231(SA) cells, MTHFD2 knockdown decreased vimentin expression also in in BT-549 cells (Supplementary Figure S3B). The cells were transfected with vimentin and MTHFD2 targeting siRNAs, scratch-wounded and wound closure was monitored for 12 hours. The results showed impaired wound healing in all studied cell lines (Fig. 5A and Supplementary Figure S3C). The most prominent decrease in relative wound confluence was observed after transient knockdown of vimentin in MDA-MB-231(SA) cells (decreased to 58%) and similar effect was also observed in response to MTHFD2 knockdown (decreased to 76% with siMTHFD2_3, 73% with siMTHFD2_7). In BT-549 cells MTHFD2 knockdown resulted in stronger decrease in wound confluence (siMTHFD2_3 71%, siMTHFD2_7 74%) than vimentin knockdown (86%). The effect of MTHFD2 depletion on cell proliferation was confirmed also in BT-549 and MDA-MB-231 cells (Sipplementary Figure S3D). Furthermore, the effect of MTHFD2 knockdown on cell invasion was studied in the most migratory cell line in this panel, MDA-MB-231(SA). Consistent with the results from wound healing assay, knockdown of vimentin led to the most dramatic decrease in cell invasion to Matrigel matrix (54%, p<0.05) (Fig 5B) and similar effect was again observed in response to MTHFD2 knockdown (siMTHFD2_3 36%, siMTHFD2_7 46%, p<0.05 for both). These results indicate that MTHFD2 is involved in the regulation breast cancer cell motility and invasion.

### MTHFD2 knockdown results in impaired vimentin network

**Figure 6:**
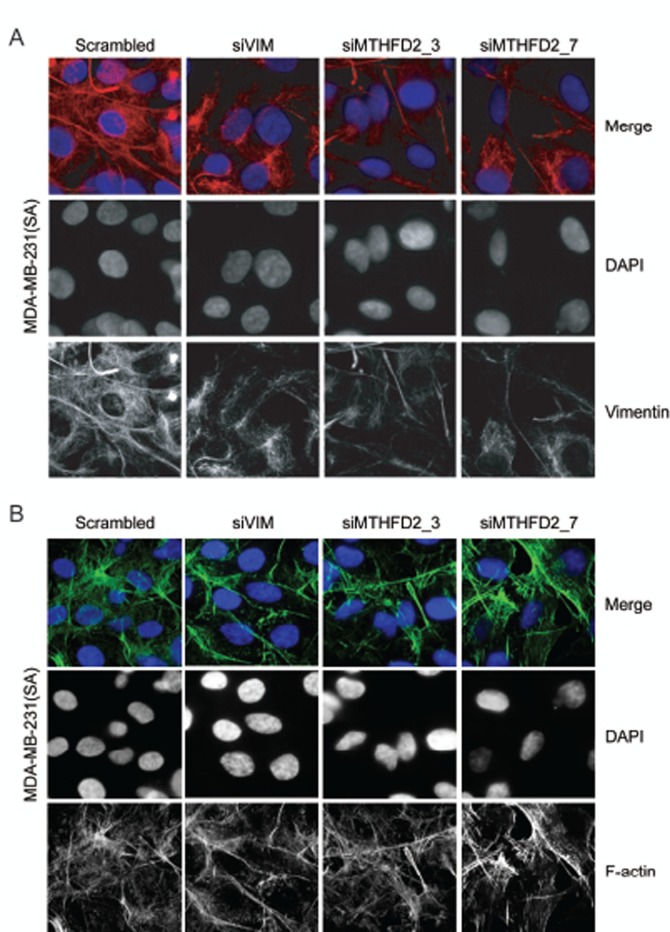
MTHFD2 knockdown disrupts vimentin network formation Immunofluorescence staining of (A) vimentin (red) and (B) F-actin (green) in MDA-MB-231(SA) cells after transient knockdown of MTHFD2. DAPI staining (blue) was used to visualize the nuclei (63x magnification).

A study by Helfand *et al.* shows that an organized vimentin network is required for cell motility [34]. The morphological changes in vimentin network induced by MTHFD2 silencing were studied in MDA-MB-231(SA) cells. After 72 hour transient silencing of vimentin or MTHFD2, vimentin network was clearly weaker and deformed compared to control cells (Fig 6A). To find out whether depletion of MTHFD2 affects cell cytoskeleton, MTHFD2 deficient MDA-MB-231(SA) were stained for F-actin expression (Fig 6B). The results indicated that while MTHFD2 depletion caused a defect in vimentin organization, it did not alter the overall cell cytoskeleton visualized by F-actin distribution.

### MTHFD2 depletion reduces N-cadherin expression

**Figure 7:**
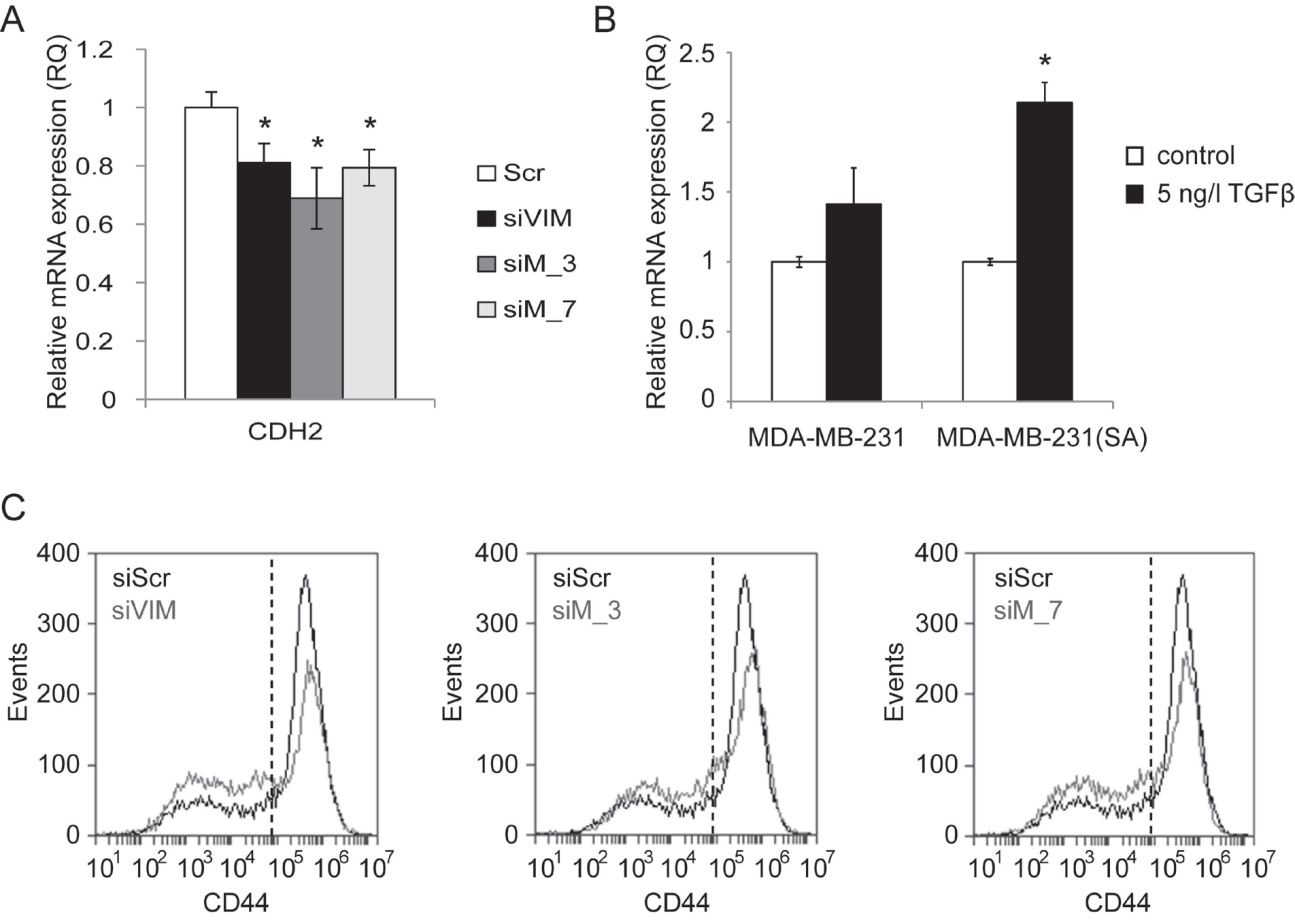
MTHFD2 depletion reduces N-cadherin mRNA expression MTHFD2 expression is induced by TGF-β and MTHFD2 knockdown reduces stem cell properties of MDA-MB-231(SA) cells. A: A qRT-PCR analysis of N-cadherin (CDH2) mRNA expression after transient MTHFD2 knockdown (*p<0.05). B: The expression of MTHFD2 mRNA was analyzed in untreated and TGF-β stimulated (5 ng/l) MDA-MB-231 and MDA-MB-231(SA) cells (*p<0.05). C: The fraction of CD44^+^ cells in MDA-MB-231(SA) cells transfected with either scrambled siRNA (siScr), vimentin targeting siRNA (siVIM) or MTHFD2 targeting siRNA (siMTHFD2). The cells were stained with CD44 antibody and the fluorescence intensities were identified with FACS analysis.

In order to study if MTHFD2 regulates other EMT related genes than vimentin, the mRNA expression of N-cadherin (CDH2), ZEB1, ZEB2 and SLUG was analysed in MDA-MB-231(SA) cells in response to either 72 h MTHFD2 or vimentin depletion. The mRNA expression of mesenchymal marker N-cadherin was decreased in response to either MTHFD2 or VIM silencing (siVIM: 81%, siMTHFD_4: 69%, siMTHFD_7: 79%; compared to scrambled control siRNA, p<0.05 for all) (Fig 7A). In addition, the mRNA expression of transcription factors ZEB1, ZEB2 and SLUG, all known to regulate E-cadherin and participate in EMT, was reduced in response to either MTHFD2 or VIM depletion, although the changes were not significant (Supplementary Figure S4). Taken together, these results indicated that in addition to vimentin, MTHFD2 depletion reduced N-cadherin expression but did not significantly modulate ZEB1, ZEB2 and SLUG transcription in MDA-MB-231(SA) breast cancer cells.

TGF-β induces MTHFD2 expression in breast cancer cells. Since TGF-β has been implicated in regulation of vimentin and promoting cell migration [21, 24], we studied whether stimulation of metastatic breast cancer cells with TGF-β affects MTHFD2 expression. Therefore, parental MDA-MB-231 and MDA-MB-231(SA) cells were stimulated with TGF-β, and MTHFD2 mRNA expression was analyzed with qRT-PCR. The results showed elevated MTHFD2 mRNA in response to TGF-β stimulation in both cell lines compared to untreated control cells (Fig 7B).

MTHFD2 knockdown reduces stem cell marker CD44. In addition to vimentin and TGF-β, the transmembrane glycoprotein CD44 has been implicated in cancer cell migration and invasion [35-37]. CD44 is also a biomarker of breast cancer tumor propagating cancer stem cells (CSCs); chemotherapy resistant cells with metastatic activity capable of forming other cancer cell populations [38-41]. The effect of MTHFD2 and vimentin knockdown on the expression of CSC marker CD44 in MDA-MB-231(SA) cells was studied with fluorescence-activated cell-sorting analysis. The fractions of CD44^low^ and CD44^high^ cells in response to MTHFD2 and vimentin knockdown are shown in Fig. 7B. Both MTHFD2 and vimentin silencing reduced the amount of CD44^high^ MDA-MB-231(SA) cells. These results indicated that MTHFD2 silencing reduced cancer stem cell properties in breast cancer cells.

### MTHFD2 depletion sensitizes breast cancer cells to methotrexate

**Figure 8:**
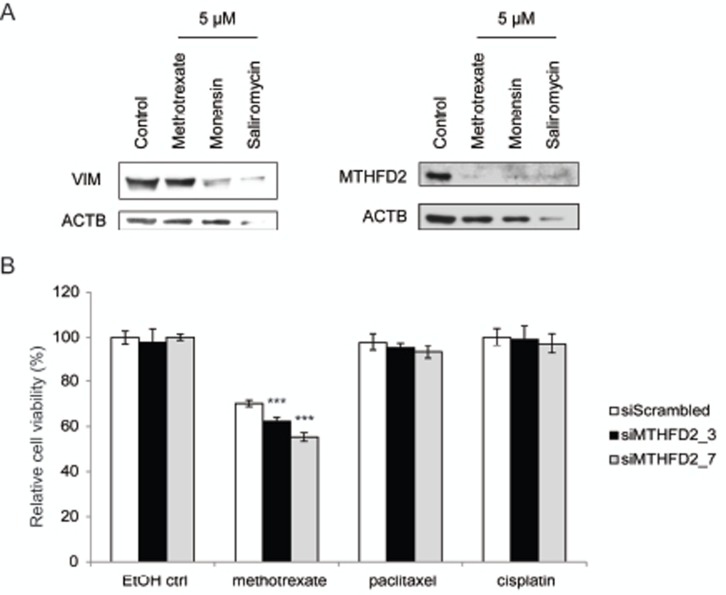
MTHFD2 knockdown sensitizes breast cancer cells to methotrexate A: Western blot analysis of MTHFD2 and vimentin expression in response to 5μM methotrexate, monensin and salinomycin. B: Analysis of cell viability in response to MTHFD2 knockdown and treatment with 0.5 μM methotrexate, cisplatin and paclitaxel (***p < 0.005).

MTHFD2 knockdown in mesenchymal type breast cancer cells induced changes indicating a switch towards more epithelial phenotype. MTHFD2 acts in one-carbon metabolism in the folate pathway. Interestingly, this pathway can be therapeutically targeted with methotrexate [42]. Previous studies indicate that salinomycin promotes differentation in breast and prostate cancer cells and monensin in prostate cancer cells [43-45]. To find out whether methotrexate, monensin or salinomycin modulate MTHFD2 or vimentin expression in breast cancer cells, MDA-MB-231(SA) cells were exposed to 5μM concentrations of these agents for 72 hours. Western blot analysis confirmed that these agents reduced vimentin as well as MTHFD2 expression, although salinomycin resulted in cell death at this concentration (Fig 8A). This indicated that in addition to decreasing vimentin expression these pharmacological agents also downregulated MTHFD2 expression, which further validated our results on the connection between vimentin and MTHFD2 in breast cancer cells.

Cancer cells have been suggested to gain resistance to conventional cancer therapeutics through induction of EMT. To study whether MTHFD2 knockdown sensitizes breast cancer cells to antineoplastic agents, MDA-MB-231(SA) cells were transfected as previously described, treated with 0.5 or 1 μM methotrexate, cisplatin or paclitaxel for 24 hours followed by measurement of cell viability. The raw values were compared to the non-treated scrambled siRNA transfected control. The results indicated that MTHFD2 silencing potentiated the anti-proliferative effect of 0.5 μM methotrexate (Fig 8). Interestingly, MTHFD2 inhibition had a significant additive effect (siM_3: 38%; p<0.005 and siM_7: 45%; p<0.005) only with methotrexate and not with paclitaxel or cisplatin, suggesting a putative combinatorial anti-proliferative response to folate pathway interference. However, at 1 μM methotrexate concentration, MTHFD2 silencing did not induce an additive anti-proliferative effect, indicating that high methotrexate concentration alone is sufficient to block the anti-neoplastic benefit resulting from MTHFD2 depletion (Supplementary Figure S5).

## DISCUSSION

Vimentin is a key regulator of breast cancer cell migration and a marker for mesenchymal subtype, characteristic of cancer cells that have undergone epithelial–mesenchymal transition (EMT). Despite the clinical relevance, very little is known about vimentin regulation in cancer. In this study, a high-throughput RNAi screening approach was undertaken to identify novel vimentin regulators among ~600 cancer relevant genes in cultured breast cancer cells. The screens were done in metastatic breast cancer cells expressing vimentin at high levels and three novel vimentin regulators - WAS/WASL interacting protein family member 2 (WIPF2), methylenetetrahydrofolate dehydrogenase 2 (MTHFD2) and ephrin type-B receptor 4 (EPHB4) were identified. Reduced vimentin protein and mRNA expression levels in *WIPF2, MTHFD2* or *EPHB4* impaired cells were confirmed also in validation experiments. Importantly, knockdown of *VIM*, *MTHFD2*, *EPHB4* or *WIPF2* did not have a strong effect on total protein expression, indicating that reduced vimentin expression was not a secondary effect caused simply by decreased cell viability.

WIPF2 has been previously described to participate in WASP-mediated organization of the actin cytoskeleton, whereas EPHB4 has been shown to promote breast cancer cell migration, invasion and survival [28, 31]. Since the biological consequences of MTHFD2 impairment in cancer cells had not been previously studied in detail, MTHFD2 was chosen for functional characterization. Moreover, our *in silico* gene expression analysis indicated an association with high MTHFD2 mRNA expression and characteristics of poor outcome in clinical breast cancer samples, indicating that MTHFD2 may promote disease progression.

Comparison of mRNA levels between normal human tissues indicated that MTHFD2 is highly expressed in blood myeloid cells as well as in hematopoietic and mesenchymal stem cells whereas among human malignancies, lymphoma and neuroblastoma showed the highest expression. Importantly, MTHFD2 mRNA levels were elevated also in clinical breast cancer samples in comparison to normal breast tissues. MTHFD2 mRNA was overexpressed especially in HER2 positive, estrogen and progesterone receptor negative breast cancer samples and was also associated with other disease characteristics (such as presence of p53 mutation and lymph node positivity) of poor outcome.

Validation of MTHFD2 mRNA expression in eight different types of human normal and malignant tissues confirmed overexpression specifically in breast cancer samples. High levels were detected particularly in breast cancer samples derived from advanced and metastatic disease. Interestingly, MTHFD2 had been previously associated as one of potential cancer biomarkers in quantitative proteomic study of cultured breast cells from normal breast tissue, primary breast cancer and metastatic breast cancer derived from the same patient [46]. In agreement with our preliminary results from human tissues, MTHFD2 is known to be expressed in tumor cells, but not in normal cells in adult mice [47]. Moreover, although MTHFD2 expression was low in other solid tumors analyzed in this study, high levels of MTHFD2 were detected in metastatic samples derived from colon cancer and renal cell carcinoma patients, indicating that MTHFD2 may promote tumor progression also in other solid tumors. Supporting these observations, elevated expression of MTHFD2 has been previously associated with increased risk of bladder cancer [30]. However, in order to get conclusive results on the putative association between high MTHFD2 expression and metastatic disease, additional studies with larger patient cohorts are required.

As a distinct biochemical feature, MTHFD2 uses NAD rather than NADP, and increases formyltetrahydrofolate content in mitochondria. Formyltetrahydrofolate is required for thymidylate synthesis and methylation reactions [48]. Interestingly, NAD dependent methylenetetrahydrofolate dehydrogenase activity was described in Ehrlich ascites cancer cells already more than fifty years ago, and twenty five years later as a general feature of transformed and non-differentiated cells [49]. Our results showing elevated MTHFD2 expression especially in cancer samples derived from patients with a metastatic disease support in general these conclusions associating NAD dependent methylenetetrahydrofolate dehydrogenase activity with transformed and non-differentiated cells. However, there seems to be clear differences in MTHFD2 expression between samples from the same as well as different cancer types since MTHFD2 expression could not be detected in all tumor samples analyzed, indicating heterogeneity in MTHFD2 expression.

Due to the identification of MTHFD2 as a modulator of vimentin expression in metastatic breast cancer cells and high expression associating with poor characteristics in clinical breast cancer samples, the cellular consequences of MTHFD2 impairment to metastasis relevant phenotypes were studied in more detail. Results from previous studies indicated that MTHFD2 promotes rapid cell growth in mice during embryonic development [47]. However, our results indicated that in highly metastatic breast cancer cells, MTHFD2 impairment did not have a significant effect on cell viability. Instead, MTHFD2 was identified as a critical regulator of breast cancer cell migration and invasion. In addition to downregulating vimentin, MTHFD2 depletion also reduced N-cadherin expression. Moreover, MTHFD2 expression was induced in response to TGF-β stimulation in breast cancer cells. This indicates that in addition to vimentin, TGF-β also regulates MTHFD2 expression. Furthermore, silencing of MTHFD2 reduced the population of CD44 cancer stem marker positive cells indicating that impairment of MTHFD2 expression reduces cancer stem cell properties in breast cancer cells. These results suggest that in addition to decreasing vimentin expression MTHFD2 depletion also reduces other characteristics typical for metastatic breast cancer cells. Interestingly, MTHFD2 expression was downregulated by several anti-neoplastic agents and treatment of MTHFD2 deficient breast cancer cells with methotrexate showed a putative additive anti-proliferative response, indicating that MTHFD2 can be therapeutically targeted and also promotes anti-proliferative effects.

In this study we show that in addition to decreasing vimentin mRNA and protein expression, MTHFD2 depletion also decreases CD44 protein expression on MDA-MB-231(SA) cells. CD44 plays a central role in the remodelling and degradation of hyaluronic acid, which leads to cell migration, invasion and cancer metastasis. A study by Cho *et al.* shows that CD44 upregulates mesenchymal markers such as vimentin and N-cadherin [50]. This supports our results showing that silencing vimentin in mesenchymal type MDA-MB-231(SA) cells decreases CD44 expression on the cell surface. In addition, vimentin targeting siRNA was more efficient in downregulating CD44 than MTHFD2 targeting siRNA and the CD44 decrease was proportional to vimentin silencing. This suggests that MTHFD2 might downregulate CD44 via vimentin. The detailed mechanism by which MTHFD2 regulates vimentin expression remains currently unknown and requires further investigation. In concordance with our results, a recent study by Selcuklu *et al.* suggests MTHFD2 as a target for miR-9, and reports that MTHFD2 knockdown reduces cell migration mimicking miR-9 overexpression [51].

This study identifies WIPF2, EPHB4 and MTHFD2 as novel regulators of vimentin expression by using a high-throughput RNAi screening approach targeting cancer relevant genes. Analysis of MTHFD2 expression showed overexpression in breast cancer samples compared to normal breast tissues. High MTHFD2 expression associated with poor clinical characteristics in breast cancer and functional characterization indicated that this enzyme is a putative regulator of breast cancer cell migration and invasion. Moreover, MTHFD2 knockdown reduced cancer stem cell properties of bone metastatic breast cancer cells. Our results suggest that mitochondrial enzyme MTHFD2 has a potential role in breast cancer progression.

## MATERIALS AND METHODS

### Cell lines and antibodies

MDA-MB-231 breast cancer cell line was from American Type Culture Collection (Manassas, VA). Bone metastatic MDA-MB-231(SA) cells were spontaneously derived from MDA-MB-231 cells during long *in vitro* culture [26]. BT-549 cells were obtained from Cell Lines Service (Eppelheim, Germany). MDA-MB-231(SA) cells were provided by Theresa Guise (Indiana University, Indianapolis, IN) and Sanna-Maria Käkönen (University of Turku, Turku, Finland). All cell lines were maintained according to distributors instructions and passaged no longer than 6 months after receive or resuscitation of frozen aliquots. Antibodies used were as follows, MTHFD2 (1:500, sc-100750, Santa Cruz Biotechnology Inc., Santa Cruz, CA), vimentin (1:1000, V6630, Sigma-Aldrich, St. Louis, MO), β-actin (1:5000, Sigma-Aldrich), Alexa Fluor 680-tagged secondary antibody (Invitrogen Inc., Carlsbad, CA) and FITC-conjugated mouse monoclonal anti-human CD44 (BD Pharmingen, San Diego, CA).

### Gene silencing

For gene knockdown, siRNAs (Qiagen, Valencia, CA) were transfected to the cells using siLentFect Lipid Reagent (Bio-Rad Laboratories, CA) in Opti-MEM (Invitrogen, Carlsbad, CA) in final concentration of 13 nM. AllStars Negative Control scrambled siRNA (siScr, Qiagen) was used as a negative control and a vimentin targeting siRNA as a positive control for vimentin knockdown (siVIM, Qiagen). Detailed siRNA information is presented in Supplementary Table S4

### LMA screening, data analysis and hit selection

The custom made siRNA library consisted of 2024 siRNAs targeting 596 genes either highly expressed in breast or prostate cancer samples, previously associated with metastasis or over-expressed in bone metastatic vs. parental MDA-MB-231 breast cancer cells [26].

A detailed description of LMA screening has been published previously [27]. Briefly, MDA-MB-231(SA) cells were transfected on 384-well plates as above. After 120 h of incubation in standard conditions, cells were lysed and lysates printed on nitrocellulose coated microarray FAST slides (Whatman Inc., Florham Park, NJ). The vimentin protein expression was detected with vimentin antibody, followed by exposure to Alexa Fluor 680-tagged secondary antibody. For total protein measurement, arrays were stained with Sypro Ruby Blot solution (Invitrogen). The slides were scanned with a Tecan LS400 (Tecan Inc., Durham, NC) microarray scanner and an Odyssey Licor IR-scanner (LI-COR Biosciences, Lincoln, NE) to detect Sypro and vimentin signals, respectively. Array-Pro Analyzer microarray analysis software (Median Cybernetics Inc., Bethesda, MD) was used for analysing data. Enrichment factor and P-value according to hypergeometric distribution were calculated using the minimum Z-score value of negative control siRNAs as a threshold. Hit limit was set to Z-score ≤ -2. To discard the effect of cell proliferation, total protein score limit was set to ≥ 0.75. The siRNAs meeting these criteria in either of the replicate screens were considered as putative vimentin expression regulating hits.

### Western blotting

Western blot analysis was done using specific antibodies mentioned above. Signal was detected with 1:4000 dilution of appropriate HRP-conjugated secondary antibodies (all from Invitrogen Molecular Probes, Carlsbad, CA) followed by visualization with the enhanced chemiluminescence reagent (Amersham Biosciences, Little Chalfont, UK) and densitometric quantification with GeneTools software (SynGene, Synoptics Ltd, UK).

### Quantitative real-time RT-PCR

Total RNA was extracted from cultured cells using RNeasy Mini kit (Qiagen) according to the manufacturer's protocol and processed to cDNA with cDNA synthesis kit (Applied Biosystems, Foster City, CA). TaqMan probes and primers were acquired from the Universal Probe Library (Roche Diagnostics, Espoo, Finland) (Supplementary Table S5). Real-time quantitative PCR (qRT-PCR) was done with ABI Prism 7900 (Applied Biosystems). Quantitation was carried out with RQ manager 1.2 software using the _ΔΔ_CT method (Applied Biosystems). Three or more replicate samples were studied for detection of target mRNA expression and β-actin used as an endogenous control. Statistical significance was determined with t-test and p-values < 0.05 were considered statistically significant.

### In silico data mining

The GeneSapiens database [52] was applied to bioinformatically explore the gene expression levels across 9783 human tissue samples. The data from normal breast (n=13) and breast cancer samples (n=957) available in the GeneSapiens database were utilized also in the *in silico* phenotype association (Miller dataset) and co-expression analyzes. The functional gene ontology annotations were analyzed for the co-expressed genes (R>0.5 and p<0.001) using Ingenuity Pathway Analysis (IPA) Software (Ingenuity Systems Inc., Redwood City, CA, USA). The survival analyzes were done using three breast cancer patient datasets extracted from Gene Expression Omnibus (GSE6532, GSE9195 and GSE12276). In these patient series, the relapse-free survival was determined from the date of diagnosis to the date of first local or distant disease recurrence, or to the date of last follow-up. The data was stratified into two groups, samples with high MTHFD2 expression (50%) and samples with low MTHFD2 (50%). For each gene, Kaplan-Meier plots were done by comparing the two sample groups using log-rank statistics. Observed differences with log-rank p-value <0.05 were considered to be statistically significant. All statistical analyzes were done using R.

### Validation of MTHFD2 mRNA expression in clinical samples

Tissue Scan human cancer cDNA panel (Origene, Rockville, MD) containing 96 samples from normal (n = 24, 3 per tissue) and malignant (n = 72, 9 per tissue) human tissues from eight tissue types (breast, colon, kidney, liver, lung, ovary, prostate and thyroid gland) was utilized to validate the expression in clinical samples. Analysis was done with qRT-PCR as above. Detailed description of sample origin is presented in Supplementary Table S6.

### Cell viability and apoptosis assays

The effects of *MTHFD2* or *VIM* silencing on cancer cell viability were assessed with CellTitre-Glo cell viability assay (Promega Inc., Madison, WI) as described [53]. Induction of caspase-3 and 7 activities was detected with homogenous Apo-ONE assay (Promega) according to the manufacturer's instructions. Fluorometric signal was quantified with Envision Multilabel Plate Reader (Perkin-Elmer, Massachusetts, MA).

### Wound healing assay

MDA-MB-231, MDA-MB-231(SA) and BT-549 cells were transfected as above and incubated on 96-well plates for 72 hours in standard conditions. A wound was scratched across each well (Wound Maker, Essen BioScience, MI) and wound closure was monitored hourly with Incucyte imaging software (Essen BioScience) for 12 hours. Wound closure was determined as percentage of wound confluence compared to respective negative control (regarded as 100%).

### Invasion assay

The effect of MTHFD2 knock-down on breast cancer cell invasion was studied as follows. Cells were plated in 30 μl of normal growth media to 18-well μ-Slides (Ibidi Gmbh, Germany) 48 hours after transfection and let to adhere for 12 hours. Cells were covered with 25% Growth Factor Reduced Matrigel (BD Biosciences, Franklin Lakes, NJ) in DMEM and incubated in +37°C for 2-3 hours to allow gelling. The slides were placed on a 4-well cell culture plate and covered with 5 ml of growth media containing 2% of FBS. The cells were allowed to invade the matrix for 4 days successively increasing the serum content of the medium to 4%, 6% and 8% FBS by changing the medium every day. Cells were stained for imaging using Calcein AM live-stain (Invitrogen) 1:500 in DMEM, 1 hour in +37°C. Imaging (~75 1.87μm stacks) was done from at least 5 different positions per each well using a Zeiss microscope (Carl Zeiss AG, Oberkochen, Germany) with a spinning disk confocal unit, 20 × objective, and SlideBook 5.0 imaging software. Z-axis projections were analyzed with the NIH ImageJ for quantitation of the invasion area.

### Immunofluorescence analysis

Immunofluorescence staining of MDA-MB-231(SA) cells was done as described [53]. Images were taken with Zeiss microscope with a spinning disk confocal unit, 20 × objective, and SlideBook 5.0 imaging software.

### Stimulation with TGF-β

For stimulation assays, cells were grown in serum deprived culture media for 24 hours before addition of TGF-β (Sigma, final concentration 5 ng/ml) in serum free media. Cells were lysed at 24 hour timepoint and RNA extracted for qRT-PCR analyzes.

### Fluorescence-activated cell-sorting analysis

MDA-MB-231(SA) cells were transfected as above. After 72 hours, samples were fixed with 4% paraformaldehyde and stained with CD44 antibody for 45 min at 41°C in the dark. Cells were washed and the fluorescence intensity was measured using Accuri C6 Flow Cytometer (BD Accuri Cytometers, Ann Arbor, MI, USA).

### Treatment with antineoplastic agents

MDA-MB-231(SA) cells were transfected as previously described. 48 hours after transfection, cells were treated with 0.5 or 1 μM methotrexate, cisplatin or paclitaxel (all diluted in EtOH) in normal growth media for 24 hours. Growth media with 1:1000 EtOH was used as control. Cell proliferation was measured with CellTiter-Glo assay (Promega) as described in the above. The raw values were compared to the non-treated scrambled siRNA transfected control. For western blot analyses, MDA-MB-231(SA) cells were treated 72 hours with 5 μM methotrexate, monensin or salinomycin (diluted in EtOH) in normal growth media.

### Statistical analyzes

Statistical analyzes were done using Student's t-test (*, P<0.05; **, P<0.01; ***, P<0.005) and Pearson correlation coefficient. Multivariate analysis was carried out with Cox´s regression. Results from cultured cells are presented as mean ±SD of at least three replicates unless stated otherwise.

## Supplementary Figures and Tables




