# Supplementary material for: High-throughput RNAi screening for novel modulators of vimentin expression identifies MTHFD2 as a regulator of breast cancer cell migration and invasion
Source: Oncotarget. 2012 Dec 17;4(1):48–63. doi: 10.18632/oncotarget.756 (PMC3702207; doi:10.18632/oncotarget.756)
Supplement: Supplementary file 1 [file oncotarget-04-048-s001.pdf]

High-throughput RNAi screening for novel modulators of vimentin expression identifies MTHFD2 as a regulator of breast cancer cell migration and invasion - Lehtinen et al

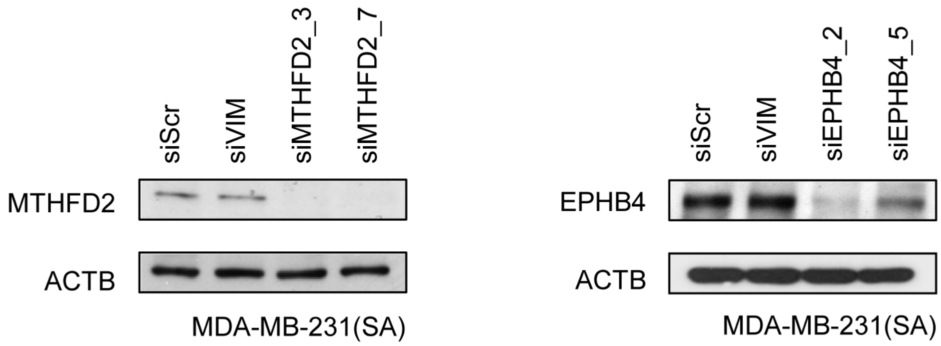

Supplementary Figure S1: Analysis of MTHFD2 and EPHB4 protein expression in MDA-MB-231(SA) cells in response to 120 h vimentin or target gene silencing.

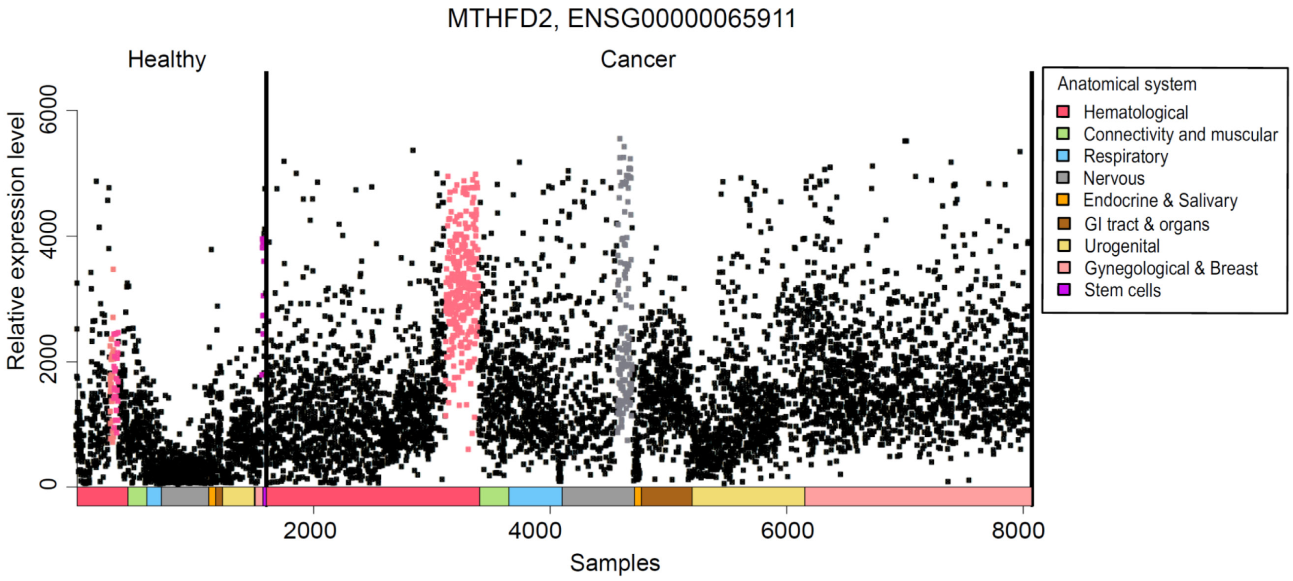

Supplementary Figure S2: MTHFD2 mRNA expression in normal and malignant tissues (n=9783) present in GeneSapiens database (www.genesapiens.com).

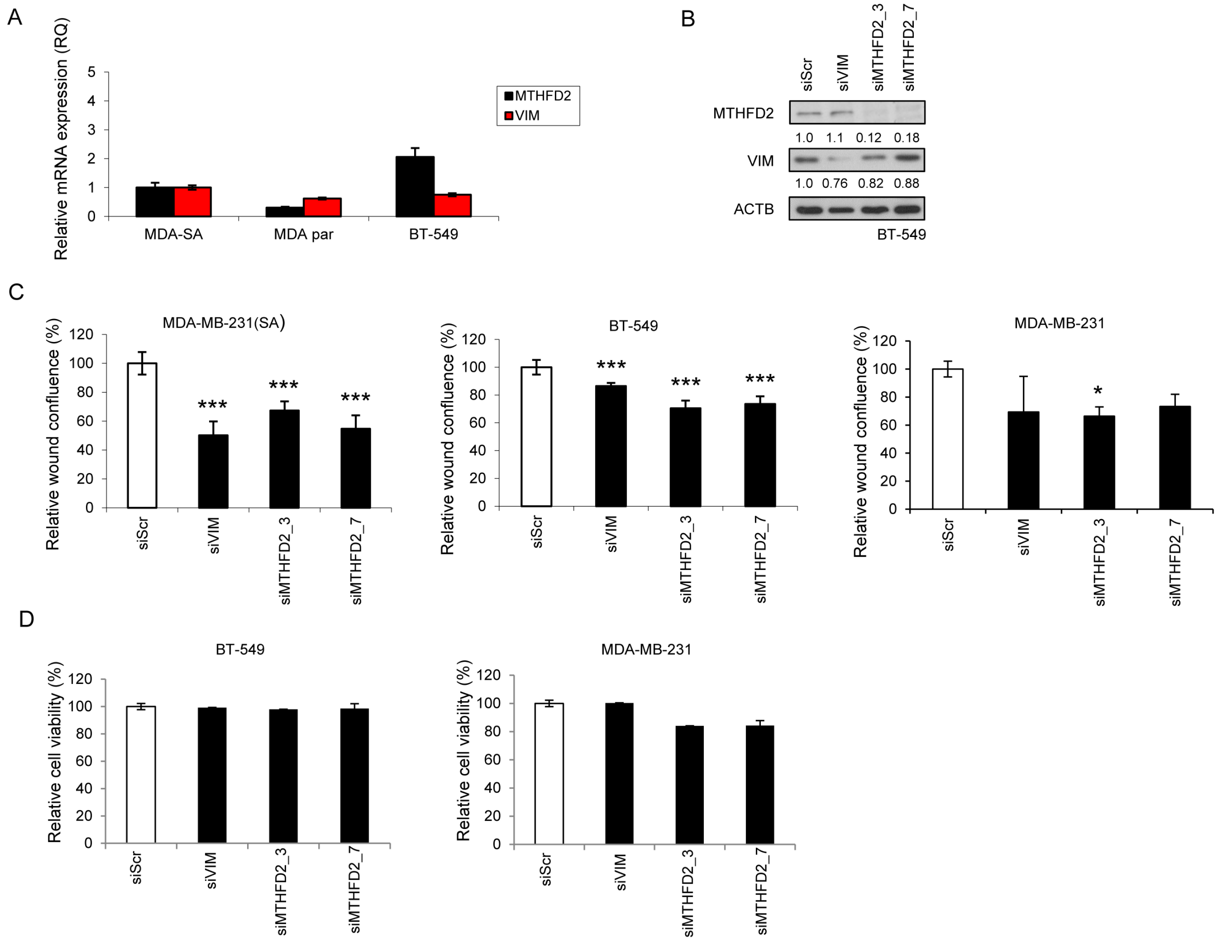

Supplementary Figure S3: A: MTHFD2 and vimentin mRNA expression in MDA-MB-231, MDA-MB-231(SA) and BT-549 breast cancer cells. B: Analysis of MTHFD2 and vimentin protein expression in BT-549 cells after 120 h transfection with either scrambled control siRNA (siScr), vimentin targeting siRNA (siVIM) or MTHFD2 targeting siRNA (siMTHFD2). C: Quantification of wound healing experiment in MDA-MB-231(SA), BT-549 and MDA-MB-231 cells. D: Analysis of cell viability in response to transient silencing of vimentin and MTHFD2 in, BT-549 and MDA-MB-231 cells. The statistically significant p-values are indicated with asterisks (* P<0.05; ** P<0.01; *** P<0.005).

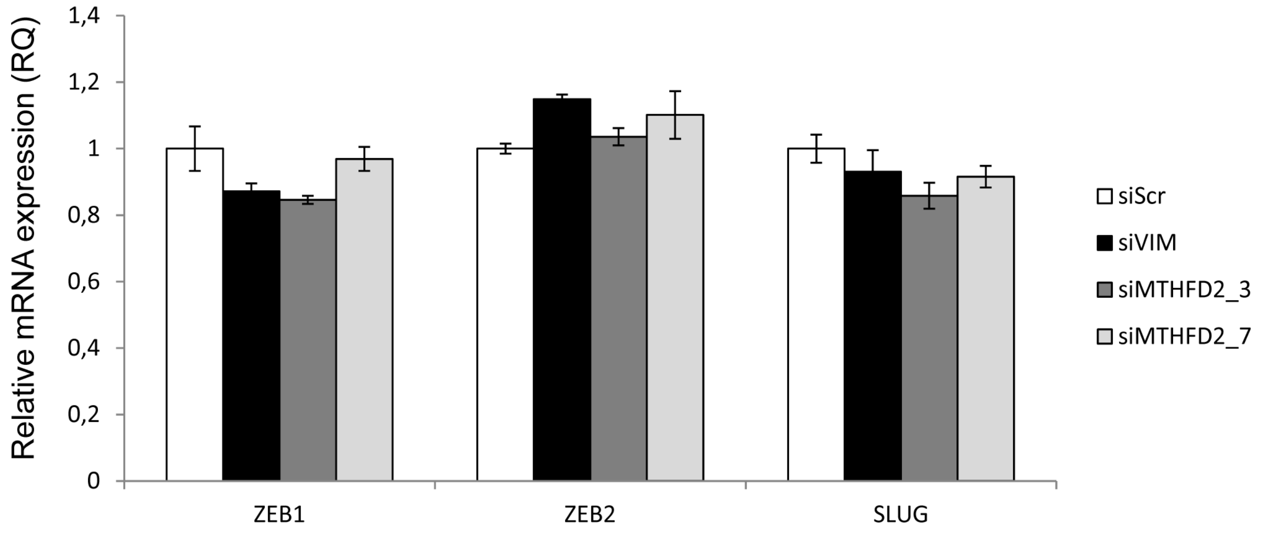

Supplementary Figure S4: Analysis of ZEB1, ZEB2 and SLUG mRNA expression after 72 h transient MTHFD2 knockdown.

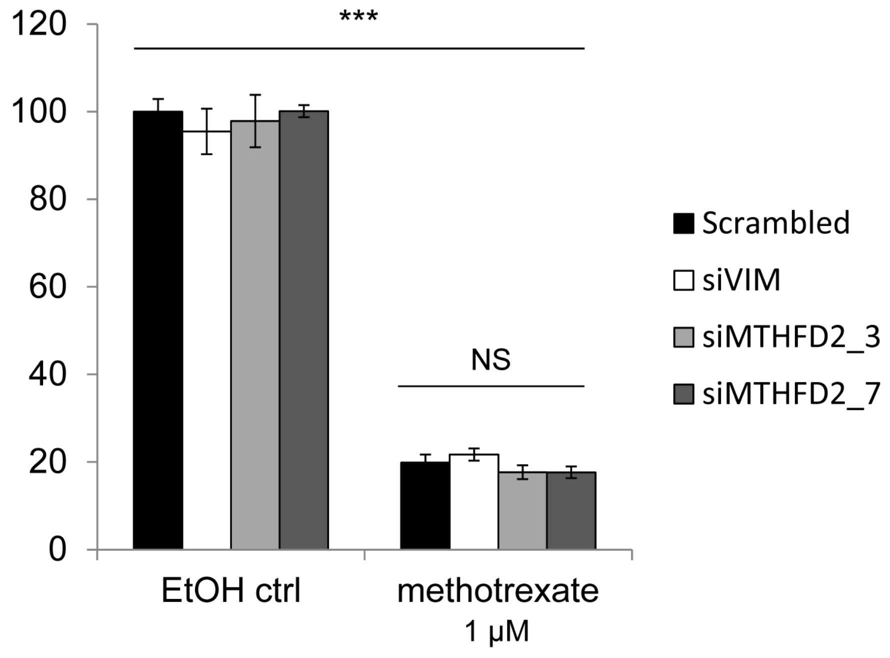

Supplementary Figure S5: Analysis of cell viability in response to MTHFD2 knockdown and treatment with 1 µM methotrexate (*p < 0.005, NS = non significant).**
